# Sex Differences in Coronary Artery Disease Characteristics Among Patients With Type 2 Myocardial Infarction

**DOI:** 10.1016/j.jacadv.2023.100795

**Published:** 2023-12-22

**Authors:** Claire Lin, Cian P. McCarthy, Reza Mohebi, Yuxi Liu, Ron Blankstein, Sean P. Murphy, Hannah Miksenas, Campbell Rogers, Daniel K. Amponsah, Paula K. Rambarat, Avanthi Raghavan, Allison Levin, Brian Ghoshhajra, Jason H. Wasfy, Sandeep Hedgire, James L. Januzzi

**Affiliations:** a Division of Cardiology, Department of Medicine, Massachusetts General Hospital, Boston, Massachusetts, USA; b Cardiovascular Division, Department of Medicine and Radiology, Brigham and Women's Hospital, Boston, Massachusetts, USA; c HeartFlow Inc, Mountain View, California, USA; d Department of Medicine, Massachusetts General Hospital, Boston, Massachusetts, USA; e Division of Cardiovascular Imaging, Department of Radiology, Massachusetts General Hospital, Boston, Massachusetts, USA; f Heart Failure and Biomarker Clinical Trials, Baim Institute for Clinical Research, Boston, Massachusetts, USA

**Keywords:** coronary computed tomography angiography, fractional flow reserve, sex-based differences, type 2 myocardial infarction

## Abstract

**Background:**

Type 2 myocardial infarction (MI) results from coronary supply and demand imbalance and has a poor prognosis. It is crucial to identify potential sex-based differences in the prevalence and nature of coronary artery disease (CAD) within this population.

**Objectives:**

The purpose of this study was to evaluate sex-based disease differences in type 2 MI among patients evaluated with coronary computed tomography angiography and fractional flow reserve.

**Methods:**

In a single-center, prospective study, patients with strictly adjudicated type 2 MI underwent coronary computed tomography angiography with fractional flow reserve.

**Results:**

Among 50 study participants enrolled, 50% were women. A similar mix of MI precipitants was present in both sexes. ST-segment depression was more common in women (64% vs 32%), while men were more likely to have T wave inversion (68% vs 36%). Women and men had comparable coronary artery calcium scores (median: 152 [Q1, Q3: 45, 762] vs 234 [Q1, Q3: 56, 422]). Prevalence of any CAD (84% vs 100%), obstructive CAD (24% vs 28%), and hemodynamically significant focal stenosis (20% vs 32%) were similar between sexes. Total plaque volume was similar between sexes, but women had significantly lower levels of low-attenuation plaque (median: 3 [Q1, Q3: 1, 7] vs 9 [Q1, Q3: 3, 14]).

**Conclusions:**

Among patients with type 2 MI, prevalence of any CAD and obstructive CAD did not differ according to sex. Total plaque volume was similar between sexes, but women had a lower volume of low-attenuation plaque (DEFINing the PrEvalence and Characteristics of Coronary Artery Disease Among Patients With TYPE 2 Myocardial Infarction Using CT-FFR [DEFINE TYPE2MI]; NCT04864119)

Type 2 myocardial infarction (MI) is a distinct MI subtype resulting from an imbalance in coronary blood supply and myocardial demand.^1^ This subtype of MI is frequently encountered in clinical practice, and studies adjudicating consecutive patients undergoing troponin testing suggest that type 2 MI has become as common as type 1 MI.^2^ Patients who experience a type 2 MI event have a high rate of recurrent cardiovascular events.^3^^,^^4^ Individuals with type 2 MI have a 3.5-fold higher relative risk of major adverse cardiovascular events than individuals without troponin elevation.^5^

Unlike type 1 MI, for which sex differences in clinical characteristics, treatment strategies, and outcomes are well documented,^6^^,^^7^ very little is known about sex differences in type 2 MI, despite women constituting a higher proportion of type 2 MI cases than type 1 MI.^8^ Limited retrospective data have suggested that women with type 2 MI have a better long-term prognosis than men with type 2 MI,^9^ but with conflicting data regarding in-hospital or short-term prognosis.^10^, ^11^, ^12^ Differences in the prevalence of coronary artery disease (CAD) may contribute to the divergent long-term prognosis for women and men with type 2 MI.^9^ Two retrospective studies observed that women with type 2 MI had a lower prevalence of obstructive disease than men.^9^^,^^11^ However, retrospective studies examining the prevalence of CAD among type 2 MI individuals have been significantly limited by reporting a broad range of CAD prevalence depending on the frequency at which a coronary evaluation is performed.^13^ Critically, in retrospective analyses, certain patients are more or less likely to receive coronary anatomic evaluation, and there is no systematic, uniform assessment of coronary anatomy with standardized testing modalities. A better understanding of sex-based differences in type 2 MI characteristics is needed, particularly regarding CAD presence and extent to provide important insights that may enable better diagnosis and management of type 2 MI.

We recently reported the primary results of the prospective defining the prevalence and characteristics of CAD among patients with type 2 MI using computed tomography-fractional flow reserve study.^14^ In this prospective trial, we used noninvasive coronary computed tomography angiography (CCTA) with fractional flow reserve (FFR_CT_) to elucidate the prevalence and characteristics of CAD in individuals experiencing type 2 MI. The DEFINE TYPE 2 MI study has an equal proportion of female and male study participants. Thus, we analyzed sex-based differences in the type 2 MI population of the DEFINE TYPE 2 MI study.

## Methods

### Study population

This is a post hoc analysis of the DEFINE TYPE 2 MI study, a prospective, single-center, investigator-initiated, observational cohort study at Massachusetts General Hospital in Boston, Massachusetts. The DEFINE TYPE 2 MI study was approved by the Mass General Brigham Institutional Review Board. Enrollment of study participants occurred between April 2021 and February 2023, where consecutive eligible inpatients with type 2 MI were approached and, if amenable, enrolled, until the target cohort of 50 adults with type 2 MI and an interpretable FFR_CT_ result was achieved. Potential study participants were initially identified through daily screening of high-sensitivity cardiac troponin T (hs-cTnT) results and via review of electronic medical records. Potential study participants with myocardial injury were then further adjudicated into diagnostic categories, including MI or myocardial injury without MI according to published diagnostic criteria.^1^ Adjudications were performed by study investigators following the universal definition of MI criteria, with uncertain cases evaluated by the principal investigator. To be categorized as type 2 MI, it was mandatory to have evidence for an imbalance in myocardial oxygen supply or demand precipitated by a medical condition or procedure and to meet the universal definition of MI, which includes a rise and/or fall in hs-cTnT concentration with at least one value above the 99th percentile combined with at least one additional symptom/sign of ischemia such as: 1) symptoms; 2) new ischemic electrocardiogram (ECG) changes or development of pathological Q waves; or 3) imaging evidence of new loss of viable myocardium. Exclusion criteria for the study included MI subtypes other than type 2 MI, hemodynamic instability preventing safe performance of CCTA, arrhythmia precluding optimal computed tomography image acquisition, or significant renal insufficiency defined as an estimated glomerular filtration rate <30 mL/min/1.73 m^2^ at the time of enrollment. Other exclusions included coronary disease factors such as prior coronary artery bypass grafting, known prior percutaneous coronary intervention of the left main coronary artery or of multiple vessels.

After obtaining informed consent, we recorded baseline patient characteristics as well as in-hospital cardiovascular testing and treatments in relation to their FFR_CT_. A coronary artery calcium (CAC) score was determined using standard approaches.^15^ Enrolled participants’ CCTA images were interpreted using the Society of Cardiovascular Computed Tomography guidelines.^16^ Additionally, blinded FFR_CT_ and coronary plaque volume (HeartFlow) analyses were performed. Medical teams responsible for the clinical care of study participants were notified of any significant cardiac findings, including obstructive CAD, and were also informed of noteworthy noncardiac findings.

The results of the DEFINE Type 2 MI study were recently published,^14^ reporting the varied modes of presentation among individuals with type 2 MI; in the study, 26% of study participants overall had obstructive CAD.

### Outcomes

Outcomes of interest for the present analysis included sex-based differences in baseline characteristics, precipitants of type 2 MI, and ECG findings. Other outcomes of interest included sex-based differences in the prevalence of obstructive CAD (defined as a stenosis of ≥70% in any epicardial vessel except the left main coronary artery, where ≥50% was considered obstructive), CAC score, prevalence of any CAD, prevalence of moderate or greater (≥50%) coronary stenosis, coronary plaque composition and burden, and prevalence of hemodynamically significant focal stenosis (FFR_CT_ of ≤0.80 1-2 cm distal to a stenosis or an occluded vessel).^17^ An FFR_CT_ of 0.50 was assigned for occluded vessels. Lastly, sex-based differences in clinical management of type 2 MI in relation to reporting of the CCTA results were evaluated, including cardiac testing and initiation/adjustment of secondary prevention therapies (aspirin, beta-blockers, statin, and angiotensin-converting enzyme inhibitors or angiotensin receptor blockers).

### Statistical analysis

Mean ± SD for normally distributed continuous variables and median (IQR) for non-normally distributed continuous variables were reported as descriptive statistics. Baseline characteristics of women vs men with type 2 MI were compared using the chi-square test. T-tests were used for normally distributed continuous variables, and the Wilcoxon rank sum was used for non-normally distributed continuous variables. CAC percentiles specific to each study participant’s age, sex, and race were calculated based on asymptomatic populations without cardiovascular disease.^18^^,^^19^ We imputed the maximum age of 84 years for which CAC percentiles exist for study participants who were 85 years of age or older.^18^ To determine plaque burden, we calculated plaque volume (total, calcified, noncalcified, and low-attenuation) percentiles specific to study participants’ age and sex. These percentiles were based on a population of 11,808 individuals who were clinically indicated to undergo CCTA.^20^ We present median values and interquartile CAC and plaque volume percentile ranges. Wilcoxon rank-sum test was used to compare the CAC scores and plaque volume data between women and men with type 2 MI. Chi-square test was used to compare the prevalence of any CAD, obstructive CAD, moderate or higher stenosis, and hemodynamically significant focal stenosis in women and men with type 2 MI. In addition, we report the percent change in the new use of medications from admission to discharge among female vs male study participants.

Given the small sample sizes, to quantify magnitude of differences in baseline characteristics between groups, we utilized standardized mean differences (SMDs). We used R software, version 4.2.2 (R Foundation for Statistical Computing) to complete all statistical analyses.

## Results

Of the 62 patients enrolled, 6 withdrew consent, and 6 did not have optimum image quality for FFR_CT_ analysis. Of the 6 uninterpretable CCTA scans, 4 were excluded due to misalignment artifact, 1 due to motion artifact, and 1 due to pixel spacing; therefore, the study population consisted of 50 patients with type 2 MI that had interpretable CCTA scans and FFR_CT_ results. The number of days that elapsed between type 2 MI and obtainment of CCTA scan was the same for women and men (median: 3 [Q1, Q3: 2, 4]). Findings are detailed in the Central Illustration.

**Figure 1:**
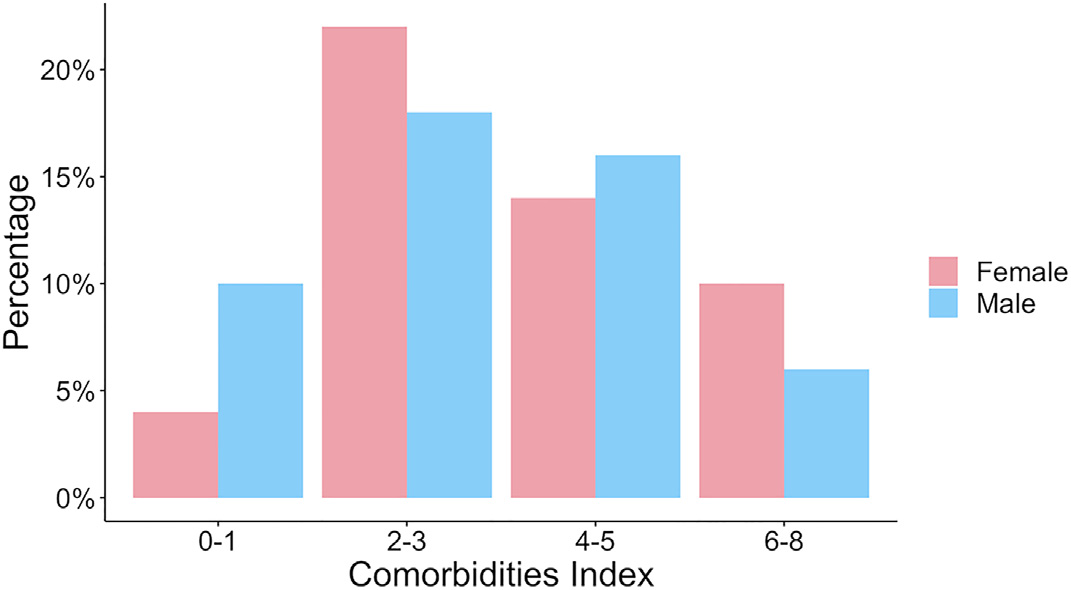
**Index Conveying Burden of Comorbidities Divided by Sex Among Study Participants With Type 2 MI** Women and men had generally comparable burdens of comorbidities. MI = myocardial infarction.

### Baseline characteristics

Of the study population, 25 (50%) were women. The baseline characteristics of women vs men study participants are displayed in Table 1. Women with type 2 MI were significantly older than men with type 2 MI, with mean ages of 72.2 ± 10.1 years vs 64 ± 11.3 years (SMD = 76.8). Both female and male study participants were mostly White (80% and 88%, respectively).

**Table 1:** Baseline Characteristics of Participants

	Female (n = 25)	Male (n = 25)	SMD (%)^a^
Age (y)	72.24 ± 10.09	64.00 ± 11.34	76.8
Race			31.9
White	20 (80.0)	22 (88.0)	
Black	4 (16.0)	3 (12.0)	
Other	1 (4.0)	0 (0.0)	
Past medical history			
Diabetes mellitus	8 (32.0)	9 (36.0)	8.5
Smoker (current or former)	11 (44.0)	15 (60.0)	32.4
Hypertension	21 (84.0)	17 (68.0)	38.1
Hyperlipidemia	18 (72.0)	13 (52.0)	42.1
Atrial fibrillation	8 (32.0)	8 (32.0)	<0.1
Prior stroke or TIA	3 (12.0)	4 (16.0)	11.5
Known CAD	3 (12.0)	2 (8.0)	13.4
Heart failure	4 (16.0)	6 (24.0)	20.1
PAD	2 (8.0)	1 (4.0)	16.9
Cancer history	5 (20.8)	4 (16.0)	12.5
Chronic kidney disease	9 (36.0)	3 (12.0)	58.6
Liver cirrhosis	0 (0.0)	2 (8.0)	41.7
Cause of type 2 MI			
Hypoxemic respiratory failure	3 (12.0)	8 (32.0)	49.8
Pneumonia	2 (66.7)	3 (37.5)	
COPD exacerbation	1 (33.3)	4 (50.0)	
RSV infection	0 (0.0)	1 (12.5)	
Tachyarrhythmia	11 (44.0)	7 (28.0)	33.8
AF or flutter	8 (32.0)	5 (20.0)	
SVT	3 (12.0)	1 (4.0)	
VT	0 (0.0)	1 (4.0)	
Hypertensive urgency/emergency	4 (16.0)	2 (8.0)	24.8
Sepsis/septic shock	1 (4.0)	0 (0.0)	28.9
Bleeding	2 (8.0)	0 (0.0)	41.7
Decompensated HF	1 (4.0)	2 (8.0)	16.9
Noncardiac surgery	3 (12.0)	3 (12.0)	<0.1
Other	0 (0.0)	3 (12.0)	52.2
Gastroenteritis	0 (0.0)	1 (4.0)	
Hypotension	0 (0.0)	1 (4.0)	
Stroke	0 (0.0)	1 (4.0)	
Clinical criteria to diagnose type 2 MI			
Chest pain	8 (32.0)	7 (28.0)	8.7
Shortness of breath	8 (32.0)	11 (44.0)	24.9
ST-segment depression	16 (64.0)	8 (32.0)	67.6
Ischemic T-wave inversions	9 (36.0)	17 (68.0)	67.6
RWMA on TTE	1 (4.0)	4 (16.0)	40.8
Symptoms only	1 (4.0)	3 (12.0)	29.8
Asymptomatic ECG changes or RMWA on TTE	11 (44.0)	10 (40.0)	8.1
Both symptoms and ECG changes and/or RWMA on TTE	13 (52.0)	12 (48.0)	8.0

Values are mean ± SD or n (%).

AF = atrial fibrillation; TIA = transient ischemic attack; CAD = coronary artery disease; HF = heart failure; MI = myocardial infarction; PTCA = percutaneous transluminal coronary angioplasty; PCI = percutaneous coronary intervention; CABG = coronary artery bypass graft; COPD = chronic obstructive pulmonary disease; SVT = supraventricular tachycardia; RWMA = regional wall motion abnormalities; RSV = respiratory syncytial virus; TTE = transthoracic echocardiogram; VT = ventricular tachycardia.

a Standardized mean difference (SMD): nonsignificant: SMD <20%; small significance: 20% ≤ SMD <50%; moderate significance: 50% ≤ SMD <80%; large significance: SMD ≥80%.

Diabetes, hypertension, hyperlipidemia, and chronic kidney disease were common in both women and men and present at similar rates. A high proportion of all study participants were current or former smokers (44% of women and 60% of men; SMD = 32.4). A small, similar proportion of women and men had known history of CAD (3 [12%] vs 2 [8%]), but no participants had a known history of obstructive CAD. Overall, women and men had similar median indexes conveying the burden of comorbidities (3 [Q1, Q3: 2, 4] vs 3 [Q1, Q3: 2, 5]) as displayed in Figure 1.

**Figure 2:**
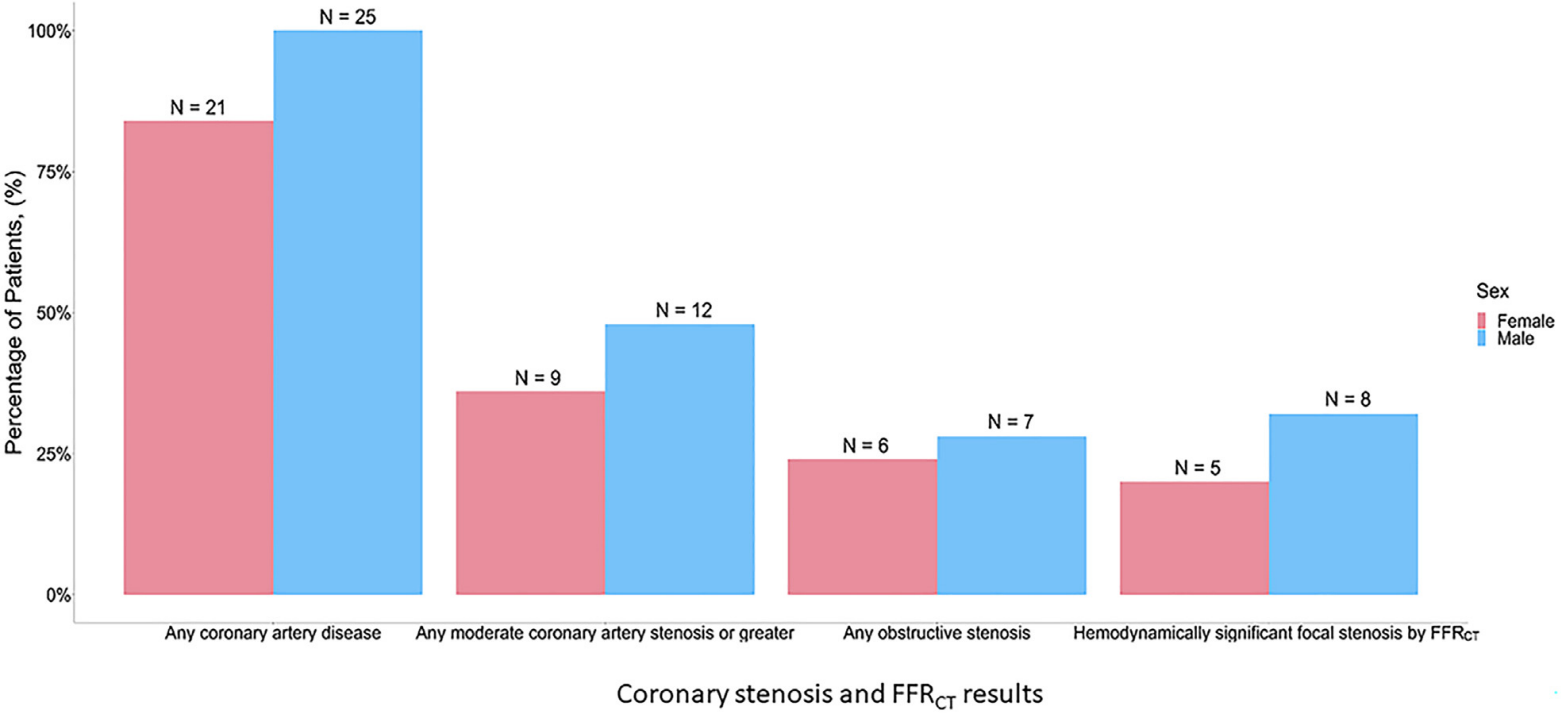
**Degree of Coronary Artery Stenosis and FFR**_**CT**_**Results in Women vs Men With Type 2 MI** Findings were generally similar between sexes. MI = myocardial infarction.

Causes of type 2 MI were similar among women and men (Table 1). In diagnosing type 2 MI, there were notable differences in ECG findings: ischemic ST-segment depression occurred more frequently in women (64% vs 32%; SMD = 67.6), while women were less likely to display T wave inversion on their ECGs compared to men (36% vs 68%; SMD = 67.6). Among the 3 individuals with acute heart failure as a cause of their type 2 MI, all had T-wave inversions in an ischemic pattern, and 2 had shortness of breath. Initial and peak hs-cTnT values were similar between women and men, although female hs-cTnT values were comparatively lower in keeping with adjusted percentiles associated with each sex. The initial median hs-cTnT concentration for women was 36 (Q1, Q3: 17, 91), while for men, it was 60 (Q1, Q3: 38, 124). Peak median hs-cTnT concentration for women was 61 (Q1, Q3: 37, 175) and 109 for men (Q1, Q3: 50, 159).

### Coronary artery stenosis

Sex-based differences in CAD are detailed in Figure 2. Prevalence of any CAD was similarly high in women and men (84% vs 100%). Women and men had a comparable prevalence of moderate or higher stenosis (9 [36%] vs 12 [48%]). Obstructive CAD, defined as stenosis of ≥70% in any epicardial vessel except left main coronary artery stenosis, where ≥50% was considered obstructive, was observed at a similar frequency in both women and men (24% vs 28%). Following the obtainment of the CCTA, 7 participants (14%) underwent invasive coronary angiography.

**Figure 3:**
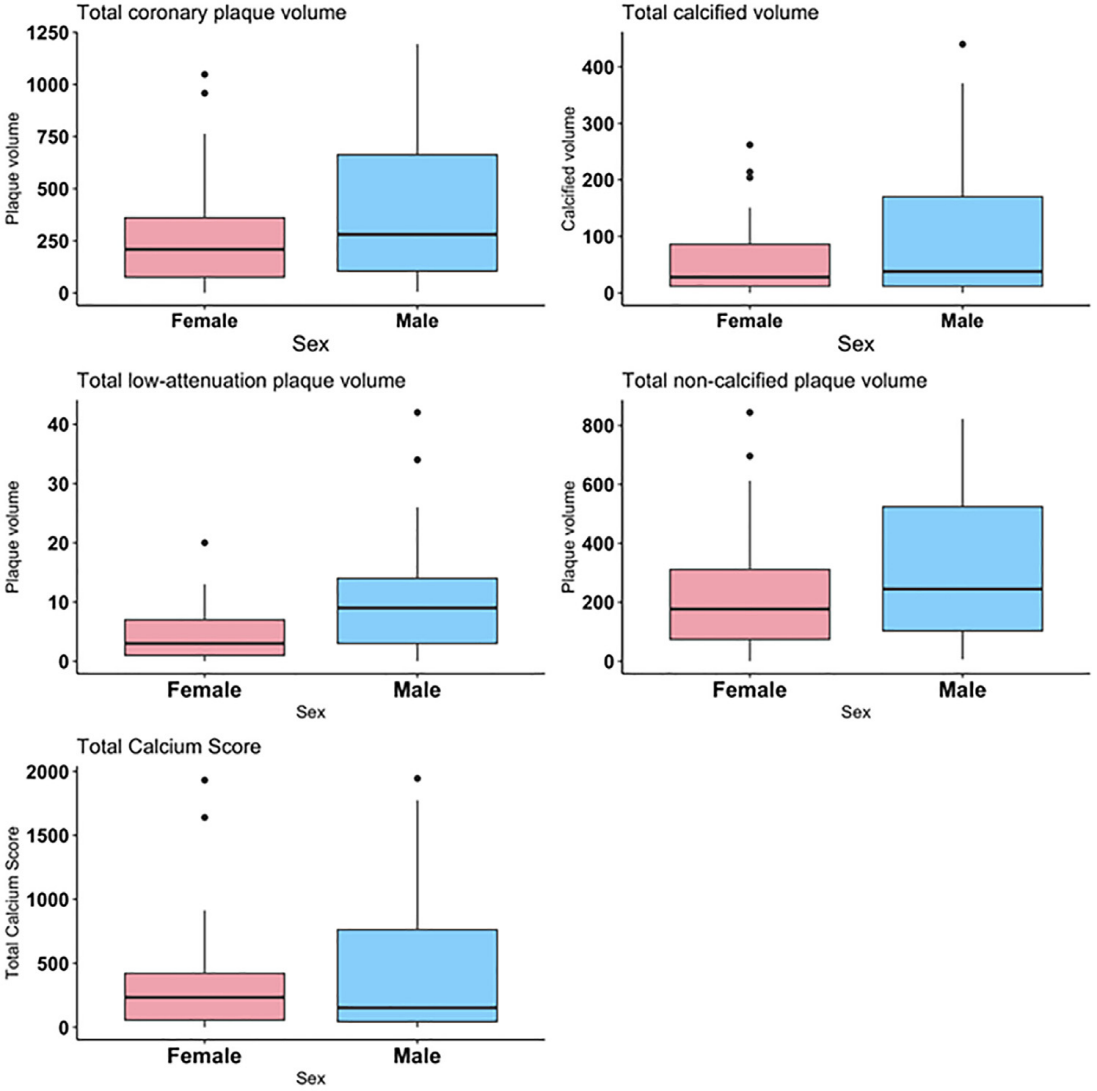
**Coronary Artery Plaque Characteristics Divided by Sex Among Study Participants With Type 2 MI** Women had significantly lower levels of low-attenuation plaque than men, while other characteristics were similar between sexes. MI = myocardial infarction.

### FFR_CT_ results

Results of FFR_CT_ analyses are also shown in Figure 2. Most participants had at least one coronary vessel with a distal segment FFR_CT_ value ≤0.80, with a similar frequency for both women and men (60% vs 76%). However, when interpreting the FFR_CT_ results in relation to the presence and location of coronary stenosis, hemodynamically significant focal stenosis was less common but also occurred equally in women and men (20% vs 32%).

Of the 21 total participants (42%) with moderate stenosis or greater (≥50%), hemodynamically significant focal stenosis was present in 13 total participants (62%), with similar frequencies of occurrence in both women and men (8 [32%] vs 5 [20%]).

### Coronary artery plaque characteristics

Characteristics of coronary artery plaque are detailed in Figure 3. The total coronary plaque volume was similar between women and men (median: 209 [Q1, Q3: 76, 361] vs 281 [Q1, Q3: 105, 663]), but coronary plaque composition differed: women had significantly lower levels of low-attenuation plaque (median: 3 [Q1, Q3: 1, 7] vs 9 [Q1, Q3: 3, 14]). There were similar levels of total calcified plaque volume in women and men (median: 28 [Q1, Q3: 12, 86] vs 38 [Q1, Q3: 12, 70]) as well as of noncalcified plaque volume (median: 245 [Q1, Q3: 103, 524] vs 177 [Q1, Q3: 74, 311]). Women and men with type 2 MI had similar levels of total plaque burden, as both approached a 50th percentile that was age and sex-specific and derived from 11,808 individuals that had clinical reason to undergo a CCTA (median: 47.4 [Q1, Q3: 20.5, 59.8] vs 47.6 [Q1, Q3: 21.4, 71.6]).^20^

**Figure 4:**
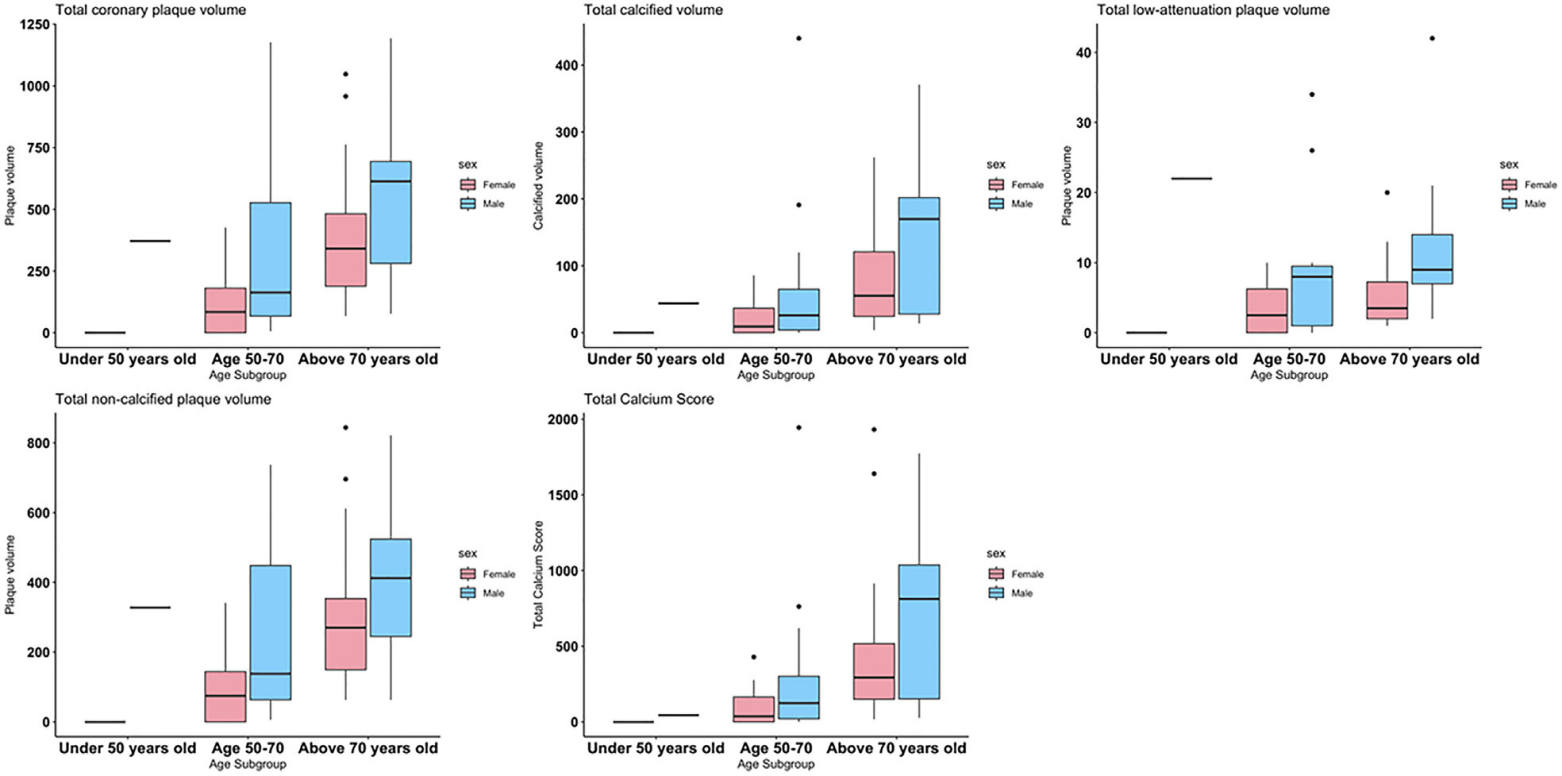
**Coronary Artery Plaque Characteristics Across Age Subgroups Divided by Sex Among Study Participants With Type 2 MI** Women generally had lower levels of plaque volume across all age subgroups. MI = myocardial infarction.

Women and men had generally similar median CAC scores (234 [Q1, Q3: 56, 422] vs 152 [Q1, Q3: 45, 762]), as shown in Figure 3. Notably, as compared with asymptomatic populations with no atherosclerotic cardiovascular disease, median CAC percentiles between women and men did not significantly differ from each other but were both above the 50th percentile (median: 75 [IQR: 48-85] vs 68 [IQR: 49-84]).

Comparisons of coronary artery plaque characteristics between women and men with type 2 MI across different age groups are shown in Figure 4. Women with type 2 MI generally had lower coronary artery plaque volumes than men with type 2 MI in each age subgroup (<50 years, 50-70 years, and above 70 years).

**Figure 5:**
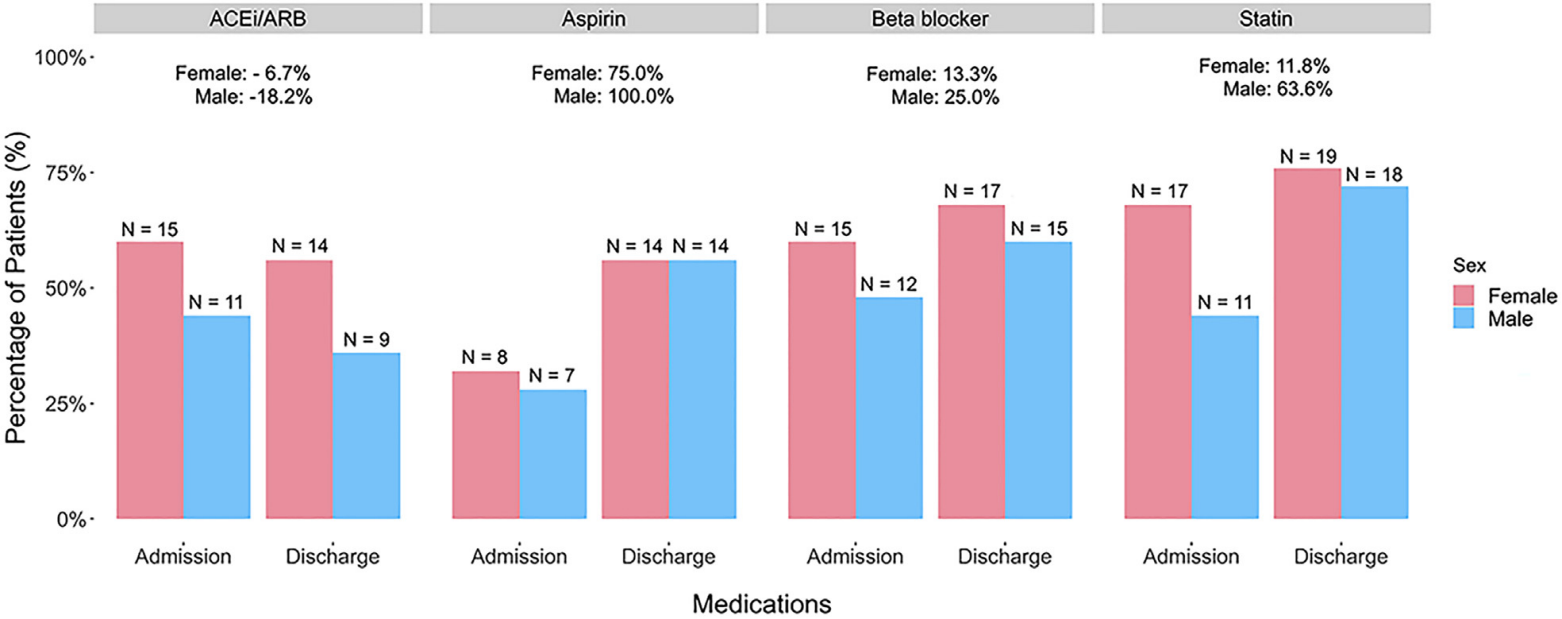
**Medications Utilization at Admission vs Discharge Divided by Sex Among Study Participants With Type 2 MI** Relative percent changes in medication use of women vs men are reported. Women and men had comparable increases in aspirin, beta-blockers, and statin utilization. ACEI = angiotensin-converting enzyme inhibitor; ARB = angiotensin receptor blocker; MI = myocardial infarction.

### Secondary treatment implementation at admission vs discharge

Medication administration in female and male study participants at time of admission and discharge, along with relative percent increases of medication use in each sex, are displayed in Figure 5. There were comparable increases in the utilization of aspirin, beta-blockers, and statins by the time of discharge for women and men with type 2 MI. For example, there was a 75% and 100% relative increase in aspirin use in female and male study participants, respectively, from hospital admission to hospital discharge; 56% of women and 56% of men were on aspirin at discharge. The proportion of women and men on beta-blockers marginally increased from admission to discharge (relative increase of 13.3% in women and 25% in men). By the time of discharge, statin prescriptions had relatively increased by 11.8% and 63.6% in women and men, respectively.

**Central Illustration:**
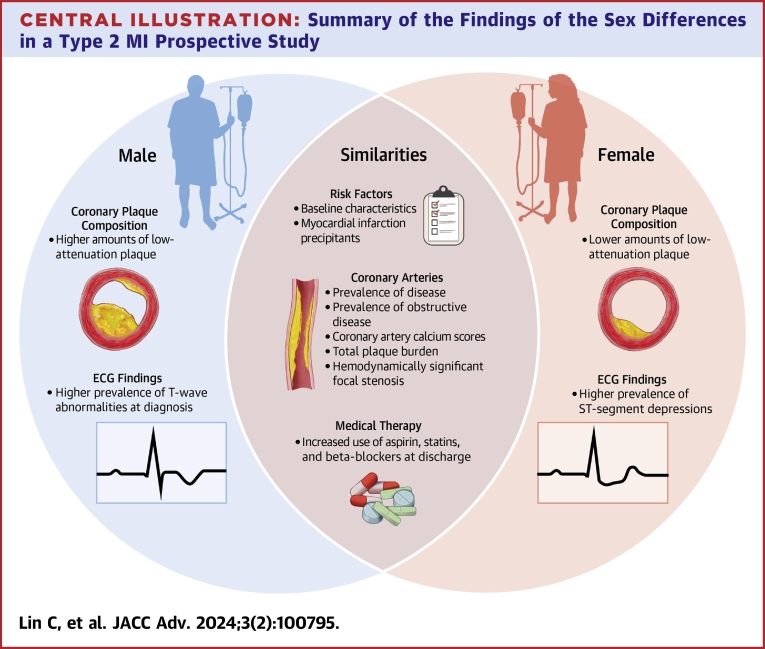
**Summary of the Findings of the Sex Differences in a Type 2 MI Prospective Study** ECG = electrocardiographic; MI = myocardial infarction.

## Discussion

In this analysis, we report sex-based similarities and differences in presentation, clinical profile, FFR_CT_ results, and clinical management of patients with strictly adjudicated type 2 MI. Women with type 2 MI had generally comparable baseline characteristics, precipitants of type 2 MI, CAD prevalence and stenosis findings, and changes in secondary medical therapy after the CCTA when compared with men, but some exceptions existed. Women with type 2 MI were older than men with type 2 MI, in keeping with general trends of cardiovascular disease manifesting in women at a later age.^21^ ECGs taken at the time of type 2 MI index admission showed women were more likely to have evidence for severe ischemia and men were more likely to display less-specific findings such as T-wave inversion. Finally, while total coronary plaque volume and plaque burden were similar between sexes, women had lower amounts of low-attenuation plaque. Understanding the nuanced presentations of type 2 MI in women vs men may aid in faster identification of type 2 MI and inform clinical management strategies.

Diagnosing type 2 MI may be challenging to secure, and a better understanding of risk factors and modes of presentation is needed. Furthermore, little is known about how type 2 MI presentation and characteristics differ between sexes, where the mechanism of MI may differ considerably. Our prior work including 359 patients adjudicated for type 2 MI reported that female study participants had fewer cardiovascular comorbidities,^11^ while a retrospective study conducted in Sweden observed that women with type 2 MI were less likely to have obstructive CAD.^9^ These previous studies lacked prospective design and adjudication of type 2 MI diagnoses. In this prospective study that leveraged careful adjudication of type 2 MI, we found generally similar baseline characteristics, atherosclerotic risk factors, and type 2 MI precipitants between female and male study participants. Notably, despite prior studies suggesting women have less CAD than men with type 2 MI, this study showed a comparable prevalence of CAD and obstructive CAD.

The prevalence of obstructive CAD in type 2 MI patients continues to be debated, with studies suggesting it ranges from 28% to 93%^22^, ^23^, ^24^ and sex-based differences remain uncertain. Similarly, little is known about sex-based differences in coronary plaque composition and burden in women and men with type 2 MI. As individuals with type 2 MI are at high risk for future atherothrombotic events, such an understanding is important to inform treatment decisions including intensity of lipid lowering. Our findings provide new insight into coronary artery plaque severity and characteristics in persons with type 2 MI based on sex. Women had similar CAC percentiles, obstructive CAD, and total coronary plaque burden as men. When compared to asymptomatic populations without atherosclerotic cardiovascular disease, women with type 2 MI had CAC scores significantly higher than the normative 50th percentile.^18^^,^^19^ However, the different coronary artery plaque compositions of women vs men with type 2 MI might imply key considerations for clinical management including intensity of lipid-lowering therapy. Considering one study reported the prognosis of men with type 2 MI to be worse than women with type 2 MI even after adjusting for baseline risk and comorbidities,^9^ it is imperative to understand not only the extent of CAD in this patient population but also how the distinct plaque compositions of women vs men factor into future cardiovascular risk.

### Study limitations

Although our study population contains an equal proportion of female and male participants and we carefully adjudicated for accuracy of type 2 MI diagnosis, it is nonetheless a small cohort, and findings are therefore limited in statistical power. Furthermore, due to the sample size, we had insufficient statistical power to determine if the prevalence of CAD and plaque characteristics differed in men and women according to the precipitating cause or mechanism of type 2 MI. The study population was also not racially diverse, which limits the generalizability of the reported findings. Additional prospective studies of the type 2 MI patient population that include a more diverse and larger number of patients would lend additional statistical power to our findings, as well as indicate any additional significant sex-based differences that did not reach statistical significance here. Although the adjudication process strictly followed the universal definition of MI criteria, there remains some subjectivity in the diagnosis of type 2 MI particularly in the absence of objective signs of ischemia and among individuals with acute heart failure, and this could have introduced selection bias during patient screening and enrollment. Finally, it is possible that stenosis severity may have been overestimated in cases of calcified plaque. Similarly, it is possible that coronary vasospasm (if present) could have led to an overestimation in stenosis severity in this population.

## Conclusions

In this prospective study of carefully adjudicated type 2 MI, there were many similarities between women and men with type 2 MI, including baseline characteristics, type 2 MI precipitants, CAD findings, and FFR_CT_ results. In addition, despite having a similar total plaque burden, women displayed lower amounts of low-attenuation plaque than men. The results of this prospective study create opportunities for furthering the understanding of differences between women and men with type 2 MI. Future studies should consider mechanistic reasons for type 2 MI and understand sex-based triggers for this increasingly common diagnosis.

PERSPECTIVES **COMPETENCY IN MEDICAL KNOWLEDGE:** Women and men with type 2 MI share similar presentations and characteristics of CAD. However, women may be of older age, display evidence of severe ischemia on ECGs, and have lower levels of low-attenuation plaque.

**TRANSLATIONAL OUTLOOK:** A better understanding of sex-based differences in type 2 MI pathophysiology may lead to specific treatment strategies for affected patients.

## Funding support and author disclosures

Dr Wasfy is supported by 10.13039/100000968American Heart Association (18 CDA 34110215); is chair of the New England Comparative Effectiveness Public Affairs Council (CEPAC); has received grant support from the American Heart Association and the National Institutes of Health; and has received past consulting fees from Pfizer and Biotronik. Dr Januzzi is supported by the Hutter Family Professorship. Dr McCarthy has received consulting income from Abbott Laboratories and Roche Diagnostics. Dr Ghoshhajra is on the Executive Committee of the Society of Cardiovascular Computed Tomography (President); has received grant support from 10.13039/501100011699Siemens Healthineers and the 10.13039/100000002National Institutes of Health; and received consulting fees from Siemens Healthineers, Philips Healthcare, and 3DR Labs (all unrelated to this work). Dr Rogers is an employee and shareholder of HeartFlow, Inc. Dr Blankstein has received research support from 10.13039/100002429Amgen Inc and 10.13039/100004336Novartis Inc; and has served as a consultant/advisory board for Caristo Inc, Elucid Inc, Hearflow Inc, Beren Therapeutics, Nanox AI. Dr Januzzi is a Trustee of the American College of Cardiology; a Director at Imbria Pharmaceuticals; an Advisor at Jana Care; has received grant support from 10.13039/100001316Abbott, Applied Therapeutics, 10.13039/100020588HeartFlow Inc, Innolife, and 10.13039/100016545Roche Diagnostics; consulting income from Abbott, Janssen, Novartis, Merck, and Roche Diagnostics; and participates in clinical endpoint committees/data safety monitoring boards for Abbott, AbbVie, Bayer, CVRx, Intercept, Pfizer, and Takeda. All other authors have reported that they have no relationships relevant to the contents of this paper to disclose.
